# Occurrence dataset from the waterbird survey of the middle and lower Huai He floodplain, China

**DOI:** 10.3897/BDJ.13.e158384

**Published:** 2025-05-22

**Authors:** Iromi Kusum Wijethunge, Jingpeng Cao, Fanjuan Meng, Zheping Xu, Qingshan Zhao, Lei Cao

**Affiliations:** 1 State Key Laboratory of Regional and Urban Ecology, Research Centre for Eco-Environmental Sciences, Chinese Academy of Sciences, Beijing, China State Key Laboratory of Regional and Urban Ecology, Research Centre for Eco-Environmental Sciences, Chinese Academy of Sciences Beijing China; 2 University of Chinese Academy of Sciences, Beijing, China University of Chinese Academy of Sciences Beijing China; 3 National Science Library, Chinese Academy of Sciences, Beijing, China National Science Library, Chinese Academy of Sciences Beijing China

**Keywords:** Sampling-event datasets, synchronous winter waterbird census, wetland

## Abstract

**Background:**

The Huai He floodplain in Anhui and Jiangsu Provinces, an important component of the East Asian-Australasian Flyway (EAAF), sustains critical wetland habitats for migratory waterbirds, including four threatened species on the IUCN Red List: critically endangered *Aythyabaeri* (Radde, 1863), endangered *Ansercygnoides* (Linnaeus, 1758) and vulnerable *Melanittafusca* (Linnaeus, 1758) and *Aythyaferina* (Linnaeus, 1758). Despite its biogeographic significance as a transitional zone between the Yangtze and Yellow River floodplains, this region remains one of China's most understudied and ecologically degraded freshwater systems. Historical pollution events and contemporary anthropogenic pressures – agricultural intensification, hydrological fragmentation and invasive species - have severely compromised wetland integrity. During mid-December 2005 and November to December 2006, standardised surveys employed fixed-radius point counts (158 sites) with the component counting method to enhance accuracy.

**New information:**

We present the first comprehensive waterbird dataset for the Anhui and Jiangsu part of the Huai He floodplain, comprising 44 species (32,517 individuals) recorded across 30 wetlands during 2005–2006 surveys. All occurrence data adhere to Darwin Core standards and are accessible via the Global Biodiversity Information Facility, providing spatial-temporal baselines for abundance and distributional data for waterbirds in this region.

## Introduction

The Huai He River Basin is one of the seven major river systems in China (Zhai et al. 2014). It lies about midway between the Yangtze and Yellow Rivers and flows in a west-east direction through Henan, Anhui and Jiangsu Provinces (He et al. 2010). It discharges into the Yangtze River after passing southwards through Hongze Hu and Gaoyou Hu (He et al. 2010). The river is 1,078 km long and has a catchment area of 174,000 km^2^ (He et al. 2010).

The Huai He River Basin is one of the most densely populated and heavily developed regions in China, characterised by an extensive network of water-related projects (Zhai et al. 2014). It also suffers from severe pollution. As the first large-scale initiative for pollution control in China, the Huai He River Pollution Control Project was launched in 1994, setting the ambitious goal of restoring the river’s water quality by the end of the 20^th^ century (Zhai et al. 2014). Although some progress was made, such as shutting down heavily polluting factories and upgrading sewage treatment infrastructure, the overall objective was not achieved by 2000 (Zhai et al. 2014). The situation further deteriorated with several acute pollution incidents in 2004, which drew international attention and highlighted the ineffectiveness of earlier efforts (Zhai et al. 2014). Due to the Basin's complex natural characteristics and intense human activity, pollution control remains an enormous challenge (Zhai et al. 2014). Water pollution in the region is exceptionally severe, leading to devastating impacts on the aquatic environment and local ecosystems (Zhai et al. 2014). Until 2013-2019, the water quality did not seem to be improving (Wang et al. 2023).

Historically, the Huai He River Basin has supported a large number of waterbirds (Cao et al. 2010). Its significance has diminished, likely because widespread control of water levels restricts habitat availability for Anatidae. In addition, pollution has severely degraded water quality, rendering it unsuitable for irrigation, industrial use or as waterbird habitat (Cao et al. 2010). Despite the presence of expansive wetlands, only a small portion – approximately 3% – of the Huai He River floodplain provided suitable habitat for Anatidae in 2005 (Cao et al. 2010).

Here, we present winter survey data collected in 2005 and 2006. The Anatidae data were reported as part of east China Anatidae population estimates and the Huai He River was not considered separately (Cao et al. 2008, Cao et al. 2010). More recent studies have reported on the entire Huai He River Basin (from December 2023 to February 2024; Li et al. (2025)) and for the Anhui section of the basin during January 2023 (Liu et al. 2024). Although the absence of detailed distribution and abundance data limits more nuanced comparisons, the overall quick comparison suggests that, while the number of species has increased, total abundance has remained stable (Liu et al. 2024). We hope that making these detailed public data available will support long-term, standardised monitoring of waterbirds in the Huai He River Basin and serve as a historical baseline for future research and comparative studies.

## General description

### Purpose

Publicly Available Occurrence Dataset from the 2005–2006 Waterbird Survey on the Middle and Lower Huai He Floodplain, China (Wijethunge et al. 2025).

## Sampling methods

### Study extent

The area surveyed consists of the lakes and reservoirs shown within Anhui and Jiangsu Provinces, extending over a distance of about 200 km upstream of Hongze Hu. All the lakes visited are connected by channels or rivers to the Huai He.

### Sampling description

The waterbird species to be counted during the survey were defined according to Waterbird Population Estimates 5^th^ Edition (Mundkur and Nagy 2012), which is the document used by the Ramsar Convention for identifying wetlands containing internationally important concentrations of waterbirds.

### Quality control

Observers generally underestimate the numbers of waterbirds present when counting large flocks (Rappoldt et al. 1985). Underestimation is also compounded by the common problem of missing birds when counting over large wetland areas.

As very large concentrations of waterbirds were not encountered during this survey, it is believed that count accuracy (identification of species and numbers) was good. It is also believed that few birds were missed at the individual count sites.

### Step description

The count was conducted by one team of two experienced counters, each using Zeiss 10 ✕ 42 Victory roof prism binoculars and Leica Televid 77 telescopes with 20–60 ✕ zoom eyepieces.

We employed a three‑tiered approach for count site selection: first, we used geo‑referenced satellite imagery to pinpoint lakes and other wetlands and loaded the coordinates into GPS units; second, we consulted personnel from the local forestry bureau and other knowledgeable locals to identify areas of high waterbird concentration and sited count points so as to cover as much of each wetland as possible; third, we selected wetland‑margin locations accessible by vehicle, hiring local transport or walking where necessary to minimise logistical difficulties. Upon arrival at each approximate location, we chose unobstructed vantage points with the sun behind us and, when wind conditions allowed, positioned ourselves downwind to stabilise monocular observations. In normal visibility, sites were spaced at roughly 3–4 km intervals (within the 2-3 km identification range), but brought nearer together under poor visibility.

On all mornings, we aimed to arrive at the first count site at about 07:00 h when it was just light enough to see birds. We were able to survey through to about 17:30 h, before it got too dark to see.

The ease with which we were able to reach wetland shores varied between lakes. In some cases, there were dykes or roads around the wetland that allowed good views of potential waterbird habitat to be obtained, whilst the shorelines of other wetlands were difficult to reach, which seriously limited our ability to adequately cover these in the available time.

## Geographic coverage

### Description

**Figure 1.**
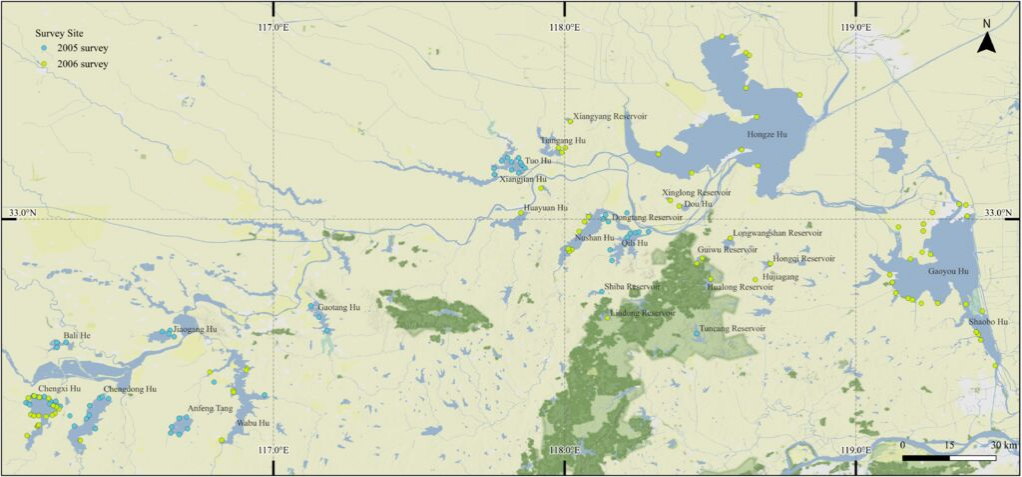
Geographic distribution of survey site.

The middle and lower Huai He floodplain, in Anhui and Jiangsu Provinces, China (Fig. 1). We conducted surveys at 158 sites across 30 lakes and reservoirs in the middle and lower Huai He River floodplain, Anhui and Jiangsu Provinces, China.

### Coordinates

32.2 and 33.7 Latitude; 116.2 and 119.5 Longitude.

## Taxonomic coverage

### Description

During the two-year sequential survey (2005–2006), 44 avian species were documented across both sampling periods. Annual census data revealed 40 species each year, comprising 15,498 individuals in 2005 and 20,445 individuals in 2006.

### Taxa included

**Table:** 

Rank	Scientific Name	
species	*Aythyabaeri* (Radde, 1863)	
species	*Anasformosa* Georgi, 1775	
species	*Anserfabalis* (Latham, 1787)	
species	*Nycticoraxnycticorax* (Linnaeus, 1758)	
species	*Larusridibundus* Linnaeus, 1766	
species	*Fulicaatra* Linnaeus, 1758	
species	*Bucephalaclangula* (Linnaeus, 1758)	
species	*Tringanebularia* (Gunnerus, 1767)	
species	*Gallinulachloropus* (Linnaeus, 1758)	
species	*Aythyaferina* (Linnaeus, 1758)	
species	*Actitishypoleucos* (Linnaeus, 1758)	
species	*Gallinagogallinago* (Linnaeus, 1758)	
species	*Anascrecca* Linnaeus, 1758	
species	*Pelecanuscrispus* Bruch, 1832	
species	*Calidrisalpina* (Linnaeus, 1758)	
species	*Casmerodiusalbus* (Linnaeus, 1758)	
species	*Botaurusstellaris* (Linnaeus, 1758)	
species	*Platalealeucorodia* Linnaeus, 1758	
species	*Anaspenelope* Linnaeus, 1758	
species	*Anasfalcata* Georgi, 1775	
species	*Anasstrepera* Linnaeus, 1758	
species	*Mergusmerganser* Linnaeus, 1758	
species	*Podicepscristatus* (Linnaeus, 1758)	
species	*Phalacrocoraxcarbo* (Linnaeus, 1758)	
species	*Aythyamarila* (Linnaeus, 1761)	
species	*Anseralbifrons* (Scopoli, 1769)	
species	*Tringaochropus* Linnaeus, 1758	
species	*Ardeacinerea* Linnaeus, 1758	
species	*Larusargentatus* Pontoppidan, 1763	
species	*Charadriusalexandrinus* Linnaeus, 1758	
species	*Egrettagarzetta* (Linnaeus, 1766)	
species	*Tachybaptusruficollis* (Pallas, 1764)	
species	*Charadriusplacidus* J.E.Gray & G.R.Gray, 1863	
species	*Anasplatyrhynchos* Linnaeus, 1758	
species	*Aixgalericulata* (Linnaeus, 1758)	
species	*Vanellusvanellus* (Linnaeus, 1758)	
species	*Anasclypeata* Linnaeus, 1758	
species	*Recurvirostraavosetta* Linnaeus, 1758	
species	*Tadornaferruginea* (Pallas, 1764)	
species	*Mergellusalbellus* (Linnaeus, 1758)	
species	*Anaszonorhyncha* Swinhoe, 1866	
species	*Tringaerythropus* (Pallas, 1764)	
species	*Ansercygnoides* (Linnaeus, 1758)	
species	*Melanittafusca* (Linnaeus, 1758)	

## Temporal coverage

**Data range:** 2005-12-09 – 2005-12-15; 2006-11-27 – 2006-12-13.

## Usage licence

### Usage licence

Other

### IP rights notes

Creative Commons Attribution Non-Commercial (CC-BY-NC) 4.0 Licence

## Data resources

### Data package title

Occurrence dataset from the waterbird survey of the middle and lower Huai He floodplain, China

### Resource link

https://doi.org/10.15468/zeuqyd

### Alternative identifiers

http://www.gbifchina.org.cn/resource?r=occurrence_dataset_waterbird_survey_huaihe_floodplain_china

### Number of data sets

1

### Data set 1.

#### Data set name

Occurrence dataset from the waterbird survey of the middle and lower Huai He floodplain, China

#### Data format

Darwin Core Event

#### Description

This dataset originates from the wintering waterbird survey of the middle and lower Huai He floodplain (Anhui and Jiangsu), China, during 2005–2006 (Wijethunge et al. 2025). It contains detailed species, abundance and geographical information.

**Data set 1.** 

Column label	Column description
eventID	An identifier for the broader dwc:Event that groups this and potentially other dwc:Events.
parentEventID	An identifier for the broader dwc:Event that groups this and potentially other dwc:Events.
samplingProtocol	The names of, references to, or descriptions of the methods or protocols used during a dwc:Event.
sampleSizeValue	A numeric value for a measurement of the size (time duration, length, area or volume) of a sample in a sampling dwc:Event.
sampleSizeUnit	The unit of measurement of the size (time duration, length, area or volume) of a sample in a sampling dwc:Event.
samplingEffort	The amount of effort expended during a dwc:Event.
eventDate	The date-time or interval during which a dwc:Event occurred.
continent	The name of the continent in which the dcterms:Location occurs.
country	The name of the country or major administrative unit in which the dcterms:Location occurs.
countryCode	The standard code for the country in which the dcterms:Location occurs.
stateProvince	The name of the next smaller administrative region than country (state, province, canton, department, region etc.) in which the dcterms:Location occurs.
county	The full, unabbreviated name of the next smaller administrative region than stateProvince (county, shire, department etc.) in which the dcterms:Location occurs.
locality	The specific description of the place.
decimalLatitude	The geographic longitude (in decimal degrees, using the spatial reference system given in dwc:geodeticDatum) of the geographic centre of a dcterms:Location.
decimalLongitude	The geographic longitude (in decimal degrees, using the spatial reference system given in dwc:geodeticDatum) of the geographic centre of a dcterms:Location.
geodeticDatum	The ellipsoid, geodetic datum or spatial reference system (SRS) upon which the geographic coordinates given in dwc:decimalLatitude and dwc:decimalLongitude are based.
coordinateUncertaintyInMetres	The horizontal distance (in metres) from the given dwc:decimalLatitude and dwc:decimalLongitude describing the smallest circle containing the whole of the dcterms:Location.
basisOfRecord	The specific nature of the data record.
dynamicProperties	A list of additional measurements, facts, characteristics or assertions about the record. Meant to provide a mechanism for structured content.
occurrenceID	An identifier for the dwc:Occurrence (as opposed to a particular digital record of the dwc:Occurrence).
individualCount	The number of individuals present at the time of the dwc:Occurrence.
organismQuantity	A number or enumeration value for the quantity of dwc:Organisms.
organismQuantityType	The type of quantification system used for the quantity of dwc:Organisms.
occurrenceStatus	A statement about the presence or absence of a dwc:Taxon at a dcterms:Location.
occurrenceRemarks	Comments or notes about the dwc:Occurrence.
scientificName	The full scientific name, with authorship and date information if known.
kingdom	The full scientific name of the kingdom in which the dwc:Taxon is classified.
phylum	The full scientific name of the phylum or division in which the dwc:Taxon is classified.
class	The full scientific name of the class in which the dwc:Taxon is classified.
order	The full scientific name of the order in which the dwc:Taxon is classified.
family	The full scientific name of the family in which the dwc:Taxon is classified.
genus	The full scientific name of the genus in which the dwc:Taxon is classified.
taxonRank	The taxonomic rank of the most specific name in the dwc:scientificName.
